# BOTULINUM TOXIN

**DOI:** 10.4103/0019-5154.60343

**Published:** 2010

**Authors:** P K Nigam, Anjana Nigam

**Affiliations:** *From the Department of Dermatology and STD, Pt. J.N.M. Medical College and Assoc. Dr. B.R.A.M. Hospital, Raipur - 492 001, India.*; 1 *From the Department of Surgery, Pt. J.N.M. Medical College and Assoc. Dr. B.R.A.M. Hospital, Raipur - 492 001, India.*

**Keywords:** *Botulinum toxin*, *Clostridium botulinum*, *clinical applications*, *adverse effects*

## Abstract

Botulinum toxin, one of the most poisonous biological substances known, is a neurotoxin produced by the bacterium *Clostridium botulinum*. *C. botulinum* elaborates eight antigenically distinguishable exotoxins (A, B, C_1_, C_2_, D, E, F and G). All serotypes interfere with neural transmission by blocking the release of acetylcholine, the principal neurotransmitter at the neuromuscular junction, causing muscle paralysis. The weakness induced by injection with botulinum toxin A usually lasts about three months. Botulinum toxins now play a very significant role in the management of a wide variety of medical conditions, especially strabismus and focal dystonias, hemifacial spasm, and various spastic movement disorders, headaches, hypersalivation, hyperhidrosis, and some chronic conditions that respond only partially to medical treatment. The list of possible new indications is rapidly expanding. The cosmetological applications include correction of lines, creases and wrinkling all over the face, chin, neck, and chest to dermatological applications such as hyperhidrosis. Injections with botulinum toxin are generally well tolerated and side effects are few. A precise knowledge and understanding of the functional anatomy of the mimetic muscles is absolutely necessary to correctly use botulinum toxins in clinical practice.

## Introduction

Botulinum toxin, also called “miracle poison,” is one of the most poisonous biological substances known.[1] It is a neurotoxin produced by the bacterium *Clostridium botulinum*, an anaerobic, gram-positive, spore-forming rod commonly found on plants, in soil, water and the intestinal tracts of animals. Scott[2] first demonstrated the effectiveness of botulinum toxin type A for the management of strabismus in humans. Subsequently, botulinum toxin was approved for the treatment of numerous disorders of spasticiy[1] and a host of other conditions. Currently it is used in almost every sub-specialty of medicine. In 2002, the FDA approved the use of Botox^®^ (Botulinum toxin-A) for the cosmetic purpose of temporarily reducing glabeller forehead frown lines.

### Biochemical aspects

*C. botulinum* elaborates eight antigenically distinguishable exotoxins (A, B, C_1_, C_2_, D, E, F and G). Type A is the most potent toxin, followed by types B and F toxin. Types A, B and E are commonly associated with systemic botulism in humans.[3] All botulinum neurotoxins are produced as relatively inactive, single polypeptide chains with a molecular mass of about 150 kDa with a high degree of amino acid sequence homology among the toxin types. The polypeptide chain consists of a heavy (H) chain and a light (L) chain of roughly 100 and 50 kDa respectively, linked by a disulfide bond.[4] The botulinum toxin neurotoxin complex is also associated with various other nontoxic proteins, which may also have hemagglutinating properties.[5]

### How botulinum toxin works

All the serotypes interfere with neural transmission by blocking the release of acetylcholine, which is the principal neurotransmitter at the neuromuscular junction. Intramuscular administration of botulinum toxin acts at the neuromuscular junction to cause muscle paralysis by inhibiting the release of acetylcholine from presynaptic motor neurons.[6] Botulinum toxins act at four different sites in the body: The neuromuscular junction, autonomic ganglia, postganglionic parasympathetic nerve endings and postganglionic sympathetic nerve endings that release acetylcholine.[5] The heavy (H) chain of the toxin binds selectively and irreversibly to high affinity receptors at the presynaptic surface of cholinergic neurones, and the toxin-receptor complex is taken up into the cell by endocytosis. The disulphide bond between the two chains is cleaved and the toxin escapes into the cytoplasm. The light (L) chain interact with different proteins (synaptosomal associated protein (SNAP) 25, vesicle associated membrane protein and syntaxin) in the nerve terminals to prevent fusion of acetylcholine vesicles with the cell membrane.[5,7] The peak of the paralytic effect occurs four to seven days after injection. Doses of all commercially available botulinum toxins are expressed in terms of units of biologic activity. One unit of botulinum toxin corresponds to the calculated median intraperitoneal lethal dose (LD_50_) in female Swiss-Webster mice.[8] The affected nerve terminals do not degenerate, but the blockage of neurotransmitter release is irreversible. Function can be recovered by the sprouting of nerve terminals and formation of new synaptic contacts; this usually takes two to three months.

Botulinum toxin induces weakness of striated muscles by inhibiting transmission of alpha motor neurones at the neuromuscular junction. This has led to its use in conditions with muscular overactivity, such as dystonia. Transmission is also inhibited at gamma neurones in muscle spindles, which may alter reflex overactivity.[9] The toxin also inhibits release of acetylcholine in all parasympathetic and cholinergic postganglionic sympathetic neurons. This has generated interest in its use as a treatment for overactive smooth muscles (for example, in achalasia) or abnormal activity of glands (for example, hyperhidrosis).[1]

The toxin requires 24-72 hours to take effect, reflecting the time necessary to disrupt the synaptosomal process. In very rare circumstances, some individuals may require as many as five days for the full effect to be observed. Peaking at about 10 days, the effect of botulinum toxin lasts nearly 8-12 weeks.

### Immunologic considerations

An estimated 5-15% of patients injected serially with earlier preparations of Botox^®^ (79-11) developed secondary nonresponsiveness from the production of neutralizing antibodies.[10]

Risk factors associated with the development of neutralizing antibodies include, injection of more than 200 units per session and repeat or booster injections given within one month of treatment. Hopefully, the new (BCB 2024) Botox^®^ has reduced immunogenicity and a lower potential for neutralizing antibody production because of its decreased protein load, though the fact is not proven in clinical trial yet.[11] In rabbit studies, no antibody formation occurred with new (BCB 2024) Botox^®^ after six months of treatment, while old (79-11) Botox^®^ caused antibody formation in all rabbits by five months.

Limited information is available on whether neutralizing antibodies resolve over time and, consequently, whether attempts at reinjection should be made after a prolonged period. An investigation is underway to determine whether injections of botulinum toxin type B are useful in patients with neutralizing antibodies to type A. Using the lowest dose of toxin necessary to achieve the desired clinical effect and avoiding reinjection within one month appear prudent in an effort to keep antibody formation as low and unlikely as possible.

### Formulation

Serotype A is the only commercially available form of botulinum toxin for clinical use, although experience is emerging with development of other serotypes: B, C, and F preparations.[12] Two preparations of botulinum toxin A exist: Dysport^®^ and Botox^®^. Unfortunately, there has been much confusion over the doses and units of potency of the two preparations.[13,14] Although doses are quoted in mouse units (which is the amount of toxin that kills 50% of a group of 18-20 g female Swiss-Webster mice), implying some standardization, Botox^®^ seems to be more potent. Recently, it has been shown that a unit of Botox^®^ is three times as potent as a unit of Dysport^®^.[14] Botox^®^ is a sterile lyophilized form of botulinum toxin type A. It is produced from a culture of the Hall strain of *C. botulinum* and purified by a series of acid precipitations to a crystalline complex containing the toxin and other proteins. The FDA approved Botox^®^ in December 1989 as an orphan drug for the treatment of strabismus, hemifacial spasms, and blepharospasm. The specific activity of Botox^®^ is approximately 20 Units/ nanogram of neurotoxin protein complex. Each vial of Botox^®^ contains 100 Units (U) of *Clostridium botulinum* type A neurotoxin complex, 0.5 milligrams of Albumin (Human), and 0.9 milligrams of sodium chloride in a sterile, vacuum-dried form without a preservative. The original batch of neurotoxin prepared by Shantz[15] in November 1979 (designated batch 79-11) constituted the original Botox^®^ formulation and was used until December 1997. It was replaced by a new neurotoxin complex batch designated BCB 2024. The new bulk batch is five to six times more potent on a weight basis. In a 100-unit vial, only 4.8 ng of neurotoxin is needed compared to 25 ng of 79-11. The new Botox^®^ is comparable in clinical efficacy and safety to the old, and a unit dose of new Botox^®^ provides an equivalent response to the same unit dose of old Botox^®^.

Dysport^®^, another formulation of botulinum toxin type A available in Europe and a few other countries, is prepared using column-based purification techniques and distributed in 500-unit vials that can be stored at room temperature. Botox^®^ and Dysport^®^ are both botulinum toxin type A preparations but are quite distinct from one another. Differences in these toxins may relate to differences in the strain of bacterium, preparation, diffusion, and potency testing.

Myobloc is a botulinum toxin type B preparation.[16]

## Preparations

### Botox cosmetic^®^

Botulinum toxin type A (BOTOX^®^; Allergan, Irvine, Calif) was the first commercially available type in the United States. Its safety is well established. The drawback is that once the contents of a vial are dissolved, the reconstituted product loses its potency. Therefore, dermatologists tend to schedule the treatments for several patients on the same day so that they can use the entire contents of the vial. This scheduling may be inconvenient for some patients, who may decide not to proceed.

### Dysport^®^ (Ipsen pharmaceuticals) (Botulinum toxin type A)

In Europe, botulinum toxin of the same serotype is marketed by another company (Dysport^®^; Speywood, United Kingdom). One unit of BOTOX^®^ has a potency that is approximately equal to 4 unit of Dysport^®^.

### Xeomin^®^

Xeomin^®^ is the third botulinum toxin type A licensed in the UK. Xeomin^®^ is an innovative Botulinum type A formulation, in which the complexing proteins have been removed by an extensive purification process from the botulinum toxin complex. In contrast to the other commercially available preparations, Xeomin^®^ contains the pure 150 kD neurotoxin. Xeomin^®^, without the complexing proteins, has the lowest content of bacterial protein of all of the available botulinum toxins and furthermore show that repeated application of Xeomin^®^, even in high doses, does not induce the formation of neutralising anti-bodies. Clinical studies have suggested that Xeomin^®^ has been found similar in its effect to Botox^®^ in clinical studies. one unit of Xeomin^®^ is equal to 1 unit of Botox^®^

### Neurobloc^®^

Neurobloc^®^ (Myobloc) is a registered trademark of Solstice Neurosciences Inc, San Francisco, Calif. It is a *Clostridium botulinum* type - B neurotoxin complex which became available in the U.K. in 2001.There is limited experience in the use of this type of toxin, and the product does not currently have approval for cosmetic use anywhere in the world. It is marketed as Myobloc^®^ Injectable Solution (botulinum toxin type B) in the United States and Canada and Neurobloc^®^ in Europe.

### Myobloc (Elan)

Myobloc^®^ (Elan), Dysport^®^ when reconstituted, has a shelf life of more than 12 months. This feature is advantageous in terms of patient scheduling. However, larger volumes of Myobloc^®^ may be needed to obtain effects similar to those of Botox^®^. Antibody formation against this product may occur more often because of its higher protein content.

### Reconstitution and storage

Botox^®^ is stored in a freezer at or below −5°C. The package insert recommends reconstitution using sterile saline without preservative; 0.9% sodium chloride is the preferred diluent. Some investigators suggest that reconstitution with sterile saline solution with preservative (0.9% benzyl alcohol) reduces microbial contamination and provides a weak local anesthetic effect.

Botox^®^ is denatured easily by bubbling or agitation; gently inject the diluent onto the inside wall of the vial and discard the vial if a vacuum does not pull the diluent in. The final dilution of Botox^®^ is mostly a matter of personal preference; 100 units commonly are reconstituted in 1-10 ml of diluent. Theoretically, more concentrated solutions reduce reliability in delivering a specific unit dose, and more dilute solutions lead to greater diffusion of the toxin.

Once reconstituted, Botox^®^ is kept refrigerated at 2-8°C. The reconstituted Botox^®^ should be used within 4 hours. One study found no loss of activity at 6 hours but a 44% loss after 12 hours and a 70% loss with refreezing at 1-2 weeks.[17] Other authors report no substantial loss of potency in a 10 U/1 ml reconstituted solution kept refrigerated for 1 month.

### How botulinum toxin is given

Botulinum toxin is injected into affected muscles or glands using a 30-gauge 1-inch needle. Doses are tailored according to the mode of use and individual patients, and the dose depends on the mass of muscle being injected: The larger the muscle mass the higher the dose required. However, lower doses may be required in patients with preexisting weakness and in females.

Toxin injections are given through hollow teflon coated needles directly into affected/overactive muscles. In localized muscle overactivity, especially, in delicate places such as strabismus, the injections are usually guided by electromyography.

### Electromyograph monitoring

Many authors[18] have chosen to administer injections under the guidance of electromyograph (EMG) monitoring. This technique involves using a 27-gauge (1.5 in) polytef-coated EMG needle connected to an EMG recorder by an alligator clip on its shaft. The patient is asked to contract the muscle in question. The injection is placed where the maximal EMG recording can be found within the muscle. This technique ensures that the injection is at the point of the muscle that is contributing most to the hyperfunctional facial line. As these injections have become routine, many centers have obtained satisfactory results without EMG guidance. Many physicians use a readily available 30-gauge insulin syringe instead. However, EMG-guided injections remain a useful adjunct in patients who have residual function after their initial injection.

### Precautions after botulinum toxin injection

As a general precaution, one should go home immediately and rest after Botox^®^. Do nothing strenuous for one or two days and refrain from laser/IPL treatments, facials and facial massage for one to two weeks after injections. This is to minimize toxins dislodging and traveling (due to increased blood circulation or direct pressure) to the surrounding muscles.

### Follow-up monitoring

The weakness induced by injection with botulinum toxin A usually lasts about three months. Hence, further injections at regular intervals are required and the interval varies widely depending on the dose and individual susceptibility. Response after the injections should be assessed both by subjective and by objective measures. Most patients treated with botulinum toxin require repeated injections over many years. Some patients who respond well initially develop tolerance to the injections due to development of neutralizing antibodies to the toxin. Patients who receive higher individual doses or frequent booster injections seem to have a higher risk of developing antibodies. Injections should therefore be given at the lowest effective dose and as infrequently as possible. Several types of antibody assay are available.[4] In clinical trials patients resistant to botulinum A have benefited from injections with other serotypes, including B, C, and F.[19]

### Clinical applications

Botulinum toxins now play a very significant role in the management of a wide variety of medical conditions, especially strabismus and focal dystonias, hemifacial spasm, and various spastic movement disorders.[9,20] Besides these, encouraging clinical reports have appeared for other uses such as headaches,[21] hypersalivation,[22] hyperhidrosis,[23] and some chronic conditions that respond only partially to medical treatment. Sometimes it can be used as an alternative to surgical intervention.[24] It seems to be a promising alternative to sphincterotomy in patients with chronic anal fissures[25] and is effective in achalasia.[26] Some autonomic disorders resulting in hypersecretion of glands like ptyalism or gustatory sweating, which often occur after surgery to the parotid gland, respond well to botulinum toxin.[23,27,28] Surprisingly, the response seems to last much longer than in conditions caused by overactivity of striated or smooth muscles.[28] The list of possible new indications is rapidly expanding [Table 1].

**Table 1:** Indications for botulinum toxin

Established indications		Tried applications							
	
Disorders of neuromuscular overactivity		Disorders of neuro-muscular overactivity		Other conditions/disorders					
		
Ophthalmic disorders	Other neuromuscular disorders	Ophthalmic disorders	Other neuromuscular disorders	Spasticity	Neuromuscular disorders	Pain	E.N.T. and oroharyngeal	Disorders of pelvic floor	Cosmetic and dermatological applications
Concomitant misalignment	Idiopathic focal dystonias	Disorders of ocular motility (nystagmus, oscillopsia)	Secondary dystonia,	Multiple sclerosis	Myokymia,	Headache (tension type, migraine, cervicogenic), neck, lower back ache	Dhtrooling of saliva, Oromandibular disorders (bruxism, Masseter hypertrophy, temporomandibular joint dysfunction)	Anismus	Wrinkles, face rejuvenation
(Primary or secondary esotropia or exotropia)	(torticollis, isolated head tremor, blepharospasm, oromandibular dystonia, lingual dystonia, laryngeal dystonia)	Thyroid disease (upper eyelid retraction, glabellar furrowing)	Tic disorders (simple tics, Tourette's syndrome, dystonic tics)	Stroke	Neurogenic tibialis anterior hypertrophy with myalgia	Myofascial pain	Pharyngeal disorders (cricopharyngeal dysphagia, closure of larynx in chronic aspiration)	Vaginismus	Browlift, crow's feet
Nonconcomitant misalignment	Other focal dystonias (writer's cramp, occupational cramps)	Therapeutic ptosis for corneal protection	Tremor (essential, writing, palatal, cerebellar)	Traumatic brain injury	Benign crampfasciculation syndrome	Tennis elbow	Achalasia,	Chronic Anal fissures	Glabellar frown
Paralytic strabismus (III, IV, VI nerves palsy, inter-nuclear ophthalmoplegia, skew deviation)	Tardive dystonia		Painful spinal myoclonus	Cerebral palsy			Laryngeal disorders (vocal fold granuloma, ventricular dysphonia, mutational dysphonia)	Detrusorsphincter dyssynergia	Frontalis frown, Bunny nose, Upper lip rhytides Pebbly chin, Naso-labial fold
Duane's syndrome	Hemifacial spasm/post-facial nerve palsy synkinesis		Parkinson's disease (freezing of gait, off period dystonia, severe constipation)	Spinal cord injury			Stuttering with glottal blocks		Platysma, Venus rings (Horizontal neck rhytides)
Restrictive or myogenic strabismus			Cephalic tetanus, stiff man syndrome, neuromyotonia				Palatal myoclonus oesophageal diverticulosis intrinsic rhinitis		Turkey neck (Vertical platysmal bands)
			Muscle stiffness, cramps, spasms						Hyperhidrosis: Palms, soles and axillae, gustatory sweating

### Dermato-cosmetological applications

Cosmetic use of BTX has skyrocketed in recent years, especially since the approval of BTX-A for treatment of glabellar lines. Until recently, Botox^®^ use was mainly confined to correct muscles of facial expression over the upper one-third of the face. Presently it's application ranges from correction of lines, creases and wrinkling all over the face, chin, neck, and chest, depressor anguli oris, nasolabial folds, mentalis, medial and lateral brow lifts, to lessen shadows on one's face and maintain a smooth outline of the jaw and cheeks from all directions, to dermatological applications such as localized axillary or palmar hyperhidrosis that is nonresponsive to topical or systemic treatment [Table 1].

### Adverse effects

Injections with botulinum toxin are generally well tolerated and side effects are few. Generalized idiosyncratic reactions are uncommon, generally mild, and transient. There can be mild injection pain and local edema, erythema, transient numbness, headache, malaise or mild nausea. Its effect diminishes with increasing distance from the injection site, but spread to nearby muscles and other tissues is possible. The most feared adverse effect is temporary unwanted weakness/paralysis of nearby musculature caused by the action of the toxin. It usually resolves in several months and in some patients in a few weeks, depending on the site, strength of the injections, and the muscles made excessively weak. Approximately 1-3% of patients may experience a temporary upper lid or brow ptosis. This results from migration of the botulinum toxin to the levator palpebrae superioris muscle. Patients often are instructed to remain in an upright position for three to four hours following injection and avoid manual manipulation of the area. Active contraction of the muscles under treatment may increase the uptake of toxin and decrease its diffusion.

The ptosis usually lasts two to six weeks. It can be treated with apraclonidine 0.5% eyedrops. This is an alpha-adrenergic agent that stimulates the Müller muscle and immediately elevates the upper eyelid. This treatment usually can raise the eyelid 1-3 mm. The treatment of one to two drops three times per day continues until the ptosis resolves. To avoid ptosis, place injections 1 cm above the eyebrow and do not cross the midpupillary line. Apraclonidine is contraindicated in patients with documented hypersensitivity. Phenylephrine 2.5% can be used alternatively. Neo-Synephrine is contraindicated in patients with narrow-angle glaucoma and in patients with aneurysms.

Weakness of the lower eyelid or lateral rectus can occur following injection of the lateral orbicularis oculi. If severe lower lid weakness occurs, an exposure keratitis may result and if the lateral rectus is weakened, diplopia results. Treatment is symptomatic.

Patients receiving injections into the neck muscles for torticollis may therefore develop dysphagia because of diffusion of the toxin into the oropharynx. When this occurs, it usually lasts only a few days or weeks. Some patients may require soft foods. Although a swallowing weakness does not herald systemic toxicity, if it is severe, patients may be at risk of aspiration. Some patients experience neck weakness, which is especially noticeable when attempting to raise the head from a supine position. This occurs after weakening of the sternocleidomastoid muscles, either from direct injection or diffusion. This is more common in women with long thin necks. Avoid these adverse effects by using the lowest effective doses and precisely placing toxin into the platysma.

Distant effects shown by specialized electromyographic tests can also occur, but weakness of distant muscles or generalized weakness, possibly due to the toxin spreading in the blood, is very rare.[29,30] however, avoid intravascular injections because diffuse spread of large amounts of toxin can mimic the symptoms of botulism.

Bruising can occur, particularly if a small vein is lacerated or a patient is taking aspirin, vitamin E, or NSAIDs. Ideally, patients should stop taking these products two weeks before the procedure. Headaches can occur after Botox^®^ injections; however, in one study by Carruthers *et al*,[31] this did not exceed the placebo group. This is thought to be due to the trauma of the injection and not something inherent in the toxin. In fact, botulinum toxin injections are extremely safe. To date, no significant long-term hazards of botulinum toxin injections have been identified in excess of placebo groups.

Other systemic side effects include an influenza-like illness and, rarely, brachial plexopathy, which may be immune mediated.[32] No severe allergic reactions have been reported, however, patient may be allergic to any of its components. Gallbladder dysfunction attributed to autonomic side effects of the toxin and a case of necrotizing fasciitis in a immunosuppressed woman with blepharospasm have been noted.[33,34]

### Contraindications to botulinum toxin injection

Botulinum toxin is contraindicated in patients afflicted with a preexisting motor neuron disease, myasthenia gravis, Eaton-Lambert syndrome, neuropathies, psychological unstability, history of reaction to toxin or albumin, pregnancy and lactating females, and infection at the injection site. Careful monitoring should be done in children as it might alter cell functions such as axonal growth.[35]

### Relative contraindications

Some medications decrease neuromuscular transmission and generally should be avoided in patients treated with botulinum toxin. These include aminoglycosides (may increase effect of botulinum toxin), penicillamine, quinine, chloroquine and hydroxychloroquine (may reduce effect), calcium channel blockers, and blood thining agents eg. warfarin or aspirin (may result in bruising).

### Therapeutic failure

Some patients do not respond to injections and, having never previously responded, are designated as primary nonresponders. Many reasons may lead to a lack of response. Patients with rhytids that are not dynamic in origin (eg, photodamage, age-related changes) do not respond. Improper injection technique or the denatured toxin may also result into therapeutic failure. Some patients may have neutralizing antibodies from prior subclinical exposure, or individual variations in docking proteins may exist.[36] Secondary nonresponders respond initially but lose the response on subsequent injections. Most of these patients may have developed neutralizing antibodies.

## Future research

Type A botulinum toxin has widened its clinical range of applications, but the risk of developing antibodies limits the repeated use of high-dose injection. Other serotypes of botulinum toxin are being investigated as useful alternatives. Botulinum toxin type F differs from type A, mainly by its lower potency, efficacy and shorter duration of action[37] and blocks a different SNARE protein as compared to type A toxin. Therefore, a combination of toxins A and F has been suggested to reduce the total units and overall antigenic dose.[38]

## Conclusion

The use of botulinum toxins has revolutionised the treatment of various ophthalmic spastic disorders, facial dystonias and periocular wrinkles. A precise knowledge and understanding of the functional anatomy of the mimetic muscles is absolutely necessary to correctly use botulinum toxins in clinical practice. Adverse effects are usually mild and transient. The most common substantive complication is excessive or unwanted weakness, and this resolves as the action of the toxin is lost. Brow ptosis, eyelid ptosis, neck weakness, dysphagia, and diplopia may occur. Knowledge of the functional anatomy and experience with the procedure help injectors avoid complications. In future, the development of new potent toxins with increasing effectiveness and duration of effect will further aid this expanding and interesting field of chemodenervation.
